# Physiological Chemistry

**Published:** 1891-06

**Authors:** John A. Miller


### Progress in Medical Science.

### PHYSIOLOGICAL CHEMISTRY.

CONDUCTED BY

JOHN A. MILLER, A. M., Ph. D.

### INFLUENCE OF MUSCULAR WORK, HUNGER, AND TEMPERATURE ON THE EXHALATION OF CARBONIC ANHYDRIDE.

V. GRANDIS (*Chem. Centr.*, 1890, I., 1069; *Jour. Chem. Soc.*, 58, 1334). The results of the author's experiments with dogs are as follows:

1. More carbon dioxide is exhaled during muscular work than when the animal is at rest, whatever the conditions as regards food are.

2. When both fasting and tired, the animals exhaled less carbon dioxide than under normal conditions, although for the first few hours after work the amount exhaled was greater.

3. The weight of the animal decreases much more rapidly after exercise than when fasting, six hours' exercise causing as great a loss of weight as when the animal fasted for four days.

4. If, after a seven days' fast, the animal is fed with double the usual amount of food, it regains its weight in three or four days, but this cannot be accomplished with animal food only. The breathing is less rapid during fasting, as also is the heart's action, especially in a warm atmosphere.

---

### THE ALKALINITY OF THE BLOOD UNDER NORMAL AND PATHOLOGICAL CONDITIONS.

E. PEIPER (*Arch. f. Pathol. Anat.*, 116, 337; *Ber. Deut. Chem. Ges.*, 23, 666,) found that the alkalinity of the blood varies within narrow limits. The blood of children is less alkaline, in reaction, than that of adults; of women, less alkaline than of men. The alkalinity of the blood is increased during *digestion*, *muscular activity* and *vomiting*. The spasms of strychnine poisoning causes a decrease.

The author states that in *leukemia*, *diabetes mellitus*, *arthritis deformans*, *chronic articular rheumatism*, *anemia*, *cancerous cachexia uremia*, and in fevers, the alkalinity of the blood is, as a rule, decreased, while in chlorosis it is increased.

### THE IDENTIFICATION OF TRACES OF ALBUMIN IN URINE CONTAINING BACTERIA.

Adolf Jolles (*Zeitsch. f. Analyt. Chem.*, 29, 407 ; *Ber. Deut. Chem. Ges.*, 23, 707,) says that by shaking the urine with "kieselguhr" (diatomatous earth) and then filtering, that the albumin can easily be identified in the filtrate by means of acetic acid and potassium ferrocyanide. In case any albumin remains on the filter, the precipitate is washed with warm caustic potash solution, and the alkaline filtrate acidified with acetic acid and tested with ferrocyanide. (We must not forget that the acetic acid and potassium ferrocyanide test is not an absolutely certain one, as *mucin* as well as albumin is precipitated by it. The treatment of the kieselguhr on the filter with warm potassium hydroxide solution is of questionable value, as we do not know whether the caustic solution has any solvent action on the bacteria themselves.—J. A. M.)

---

### ACTION OF YEAST ON THE ANIMAL AND HUMAN ORGANISM.

J. Neumayer (*Inaug. Diss. München. Hygien. Inst.; Jour. Chem. Soc.*, Vol. LX., p. 237,) finds that the various yeasts pass through the alimentary canal without suffering any change, and are not acted on by the gastric juice, and that they may be eaten without harm to the animal, provided that all fermentable substances are absent, otherwise inflammation of the stomach ensues. This injurious action is not due to the yeasts, or the principal products of their fermentative action, but to abnormal fermentation products formed at the high temperature of the body ; if the fermentation proceeds at low temperature, these injurious products are not formed at all, or only in insignificant amount. Subcutaneous injection of the yeasts causes their destruction, without, however, producing any ill effects on the animal.

---

### TRANSFUSION OF MIXTURES OF BLOOD AND SALT SOLUTION.

By J. Marshall (*Zeit. Physiol. Chem.*, 15, 62 ; *Jour. Chem. Soc.*, 60, 347). Transfusion of salt solution is now so often used in medical practice, that it is important we should have an accurate knowledge of the process of blood regeneration that follows this procedure. Rabbits were bled until convulsions were imminent, and then a solution of one part of defibrinated blood to nine parts of 0.06 sodium chloride solution was injected ; tables are given with enumeration of blood corpuscles, and percentages of hemoglobin before and at intervals after the operation. When regeneration was complete, the animals were killed, but in no case was anything abnormal found at the autopsy. The blood corpuscles reach the normal number in a few days, but the percentage of hemoglobin is not normal for some days, in one experiment not until fifteen days later. The results coincide closely with those of J. G. Otto, who used no injection, but simply watched the course of regeneration after hemorrhage in rabbits.

---

### THE INFLUENCE OF DRINKING LARGE QUANTITIES OF WATER ON THE EXCRETION OF URIC ACID.

By B. Schoendorff (*Pflueger's Archiv.*, 46, 529 ; *Jour. Chem. Soc.*, 60, 348). The result of the author's experiments is that whilst the total nitrogen is increased, the drinking of large quantities of water has practically no influence on the uric acid.

---

### A BACTERIA-KILLING GLOBULIN.

By E. H. Hankin (*Proc. Roy. Soc.*, 48, 93 ; *Jour. Chem. Soc.*, 60, 352). The results described in this paper were arrived at by the author while trying to discover the nature of the substance to which the bacteria-killing powers of the blood serum is due. Halliburton's cell-globulin—B was extracted from the lymphatic glands of an animal by sodium sulphate solution. It was found to have the power of killing anthrax bacilli, which property seems to distinguish it from fibrin ferment. This bacteria-killing power is of the same nature as that possessed by blood serum, which, therefore, probably acts in virtue of the same or of some allied substance. From his experiments, the author further concludes that, inasmuch as it is possible to obtain from the cells that are, or can become, phagocytes, a substance having bacteria-killing powers, it may be supposed that phagocytes cannot only kill microbes that they have ingested, but also do this by breaking down, and liberating their contents.

---

### BACTERIAL POISONS.

By Victor C. Vaughan (*Med. News; Popular Science News*). The author sums up the present state of our knowledge on the above subject as follows :

1. Man is attacked by the infectious diseases either through the alimentary canal or through the blood or lymph.

2. The gastric juice is a physiological guard against infection by the way of the intestines.

3. Additional guards against infection by the intestines are probably to be found in the absorbing cells of the stomach and intestines.

4. Susceptibility to the intestinal infectious diseases is increased when, for any reason, these physiological guards are defective.

5. All toxicogenic germs are dangerous when introduced into the intestines, and their capability of doing injury lies in their production of chemical poisons.

6. Many of these poisons are proteid in character.

7. These poisonous proteids most probably act by catalysis.

8. In the splitting up of complex molecules into simpler ones, heat is liberated and fever manifests itself.

9. The physiological guard against infection through the blood or lymph lies in the germicidal action of the proteids of these fluids.

10. Susceptibility to infection through the blood or lymph is increased by impoverishment of these fluids.

11. We can continue to treat consumption and other systemic diseases by the employment of liberal diet, exercise in the open air, and constitutional remedies without being unscientific in our practice.

12. Filth, without being the bearer of a specific germ, is a cause of disease.

13. Wherever man pollutes the soil about him, the air which he breathes, and the water which he drinks with his own excretions, there enteric fever will be found.

14. In their causal relation to disease, germs cannot be classified without a knowledge of the chemical changes which they induce.

15. While certain bacterial poisons can result only from the growth of certain germs, other poisons similar to one another in their action, though probably not identical, may result from any one of a number of organisms. In the former case we have such diseases as anthrax and small-pox, with their practically constant symptoms and well-marked course ; in the latter case we have such diseases as the summer diarrhea of infancy and enteric fever, with their varying symptoms.

### ACCIDENTS IN ANESTHESIA.

DR. H. C. WOOD (*Jour. Am. Med. Ass'n*), of Philadelphia, gives the following rules for the proper treatment of accidents during anesthesia :

Avoid the use of all drugs, except strychnine, digitalis, and ammonia.

Give the tincture of digitalis hypodermically.

Draw out the tongue, and raise up the angle of the jaw, and see that the respiration is not mechanically impeded.

Invert the patient briefly and temporarily.

Use forced artificial respiration promptly, and in protracted cases employ external warmth and stimulation of the surface by the dry electric brush, etc.; and, above all, remember that some, at least, and probably many, of the deaths which have been set down as due to chloroform and ether have been produced by the alcohol which had been given for the relief of the patient.

